# The surgical treatment of female stress urinary incontinence

**DOI:** 10.4103/0970-1591.65401

**Published:** 2010

**Authors:** Christopher K. Harding, A. C. Thorpe

**Affiliations:** Department of Urology, Freeman Hospital, Newcastle-upon-Tyne, UK

**Keywords:** Review, stress urinary incontinence, females

## Abstract

Urinary incontinence is a common symptom experienced by significant numbers of adult women. Stress urinary incontinence (SUI) is the most frequently encountered type and affects around 50% of incontinent females. Many affected women do not consult their doctors about this troublesome symptom perhaps based on a belief that they cannot be helped. Recent years have seen the development of several new and popular techniques for the surgical treatment of this condition and many of the “gold standard” procedures for stress incontinence have been challenged. Currently, evidence in favor of the use of sub-urethral tapes especially tension-free vaginal tapes suggests that a new standard of low morbidity and high efficacy surgical treatment for SUI has been set. This review is intended to examine all of the surgical options for the treatment of SUI and provide health care professionals with an overview of the vast array of currently available procedures.

## INTRODUCTION

Stress urinary incontinence (SUI) is a symptom, a clinical sign, and a urodynamic observation. The symptom of SUI is best defined by the International Continence Society as “involuntary leakage of urine on exertion, effort, coughing, or sneezing.[1] SUI is the most commonly diagnosed subtype of incontinence in adult women. The majority of published data suggest that around 50% of incontinent women will exhibit pure SUI with a further 30% experiencing mixed incontinence. Thus potentially large numbers of adult women with troublesome urine leakage will have a “stress component” to their incontinence. In a review of population-based studies published in 2003, Minassian *et al* found a wide range in the prevalence of incontinence reported in the literature.[2] The median prevalence of female urinary incontinence (UI) was 27.6% (range: 4.8-58.4%) and the prevalence of significant incontinence increased with age. Other risk factors included parity, obesity, chronic cough, depression, poor health, lower urinary tract symptoms, previous hysterectomy, and stroke. Despite the well-documented effect on quality of life, the authors comment that most women did not seek medical help.

These data are supported by a large pan-European study reported by O'Donnell *et al*, including a sample of almost 30,000 women assessed via a postal questionnaire.[3] They found that less than one in three women with UI had consulted their doctor and that this pattern was reproducible across all countries with little variation. It would seem therefore that despite the bothersome nature of their symptoms and the obvious effect on quality of life, the majority of women are disinclined to seek medical intervention perhaps in the belief that treatment will not be successful or that surgery for their condition is perceived as unacceptable. We present an overview of the diagnostic and therapeutic interventions for SUI designed to help health care providers in the counseling of these women prior to consideration of treatment.

## DIAGNOSING STRESS INCONTINENCE

The diagnosis of SUI is suggested from the clinical history, but recent reports have questioned the validity of this approach and recommend further investigations prior to making a diagnosis and considering treatment. Agur *et al* examined precisely this issue in a retrospective series of over 6,000 women and concluded that a diagnosis of pure SUI could be made in only 5% of women based on history alone.[4] Furthermore, around one in four women with a suggested diagnosis of SUI from the clinical history did not have SUI diagnosed following urodynamic studies (UDS) suggesting a highly significant level of inaccuracy if clinical history alone is used. This study is supported by another report with strikingly similar findings. Digesu *et al* report from a series of almost 3,500 women and state that a diagnosis of SUI based on clinical history can only be made in less than 10% of women. In addition, over 20% of those women were given alternative diagnoses following UDS.[5] These data highlight the level of inaccuracy associated with history alone and recommend further investigation prior to recommending treatment for SUI.

The diagnosis of SUI is often made following UDS, and Blaivas has suggested a classification of SUI based on observations from video urodynamics and grading SUI based on both the degree of urethral mobility and function of the urinary sphincter.[6] Urethral hypermobility is the predominant abnormality in types 1 and 2 in the Blaivas classification and is commonly due to weakness of the pelvic floor. This allows rotational descent of the bladder neck and proximal urethra during increased abdominal pressure. Type 3 SUI describes a sphincter mechanism malfunction and has numerous causes.

### Type 0

A good history of stress incontinence is obtained. During the videourodynamic study, the vesical neck and proximal urethra are closed at rest, being situated at or above the superior margin of the pubic symphysis. During stress (cough or strain) although the vesical neck and proximal urethra open, no leakage is observed.

### Type 1

The vesical neck is closed at rest, and is situated at or above the inferior margin of the pubic symphysis. During stress maneuver with increased abdominal pressure, the vesical neck and proximal urethra open and descend < 2 cm, with urinary incontinence being demonstrated.

### Type 2a

The vesical neck is closed at rest and above the inferior margin of the pubic symphysis. During stress, the vesical neck and proximal urethra descend >2 cm and urinary incontinence is demonstrated.

### Type 2b

At rest, the vesical neck is closed but is situated below the inferior margin of the pubic symphysis. During stress, there is further descent, the proximal urethra opens and urinary incontinence is demonstrated.

### Type 3

At rest, the vesical neck and proximal urethra are open, despite the absence of a detrusor contraction, there is obvious leakage of urine, which is either gravitational or associated with a minimal increase in intravesical pressure.

A useful adjunct in the diagnosis of SUI is the abdominal leak point pressure measurement, which is defined as the intravesical pressure at which urinary leakage occurs due to increased abdominal pressure in the absence of a detrusor contraction.[1] This investigation was devised by McGuire[7] and helps to define urethral function and in particular identify those women who have intrinsic sphincter deficiency from those with urethral hyper mobility. In general, those women with an abdominal leak point pressure of less than 90 cmH_2_O are diagnosed as having intrinsic sphincter deficiency (type III SUI according to the Blaivas classification).

## TREATMENTS FOR STRESS URINARY INCONTINENCE

Once the diagnosis of pure SUI or mixed incontinence with a predominant “stress component” is made with confidence then treatment modalities should be fully discussed with the affected patient and all options, both surgical and non-surgical, should be outlined. Conservative management of SUI is beyond the scope of this review but options include behavioral therapy, timed voiding, fluid management, smoking and caffeine reduction, weight loss, and other lifestyle modifications. Perhaps the strongest body of evidence for conservative treatment supports the use of pelvic floor muscle training (PFMT) or biofeedback. Wilson *et al* reviewed the current evidence for PFMT at the International Consultation on Incontinence 2005[8] and concluded that the overwhelming majority of the trials showed effectiveness of PFMT. In addition, the recommendation that PFMT should be offered, as first line therapy, to all women with stress, urge or mixed incontinence was made. Biofeedback techniques including the use of vaginal cones was also evaluated at this consultation with the conclusion that biofeedback was better than no treatment but not superior to PFMT.[8]

If conservative measures fail then surgical treatments are considered; these can be sub-divided based on four major types of procedure;

- Peri-urethral bulking agent injection
- Retropubic suspension (colposuspension, paravaginal repair)
- Sling and tape procedures (including sub-urethral tapes and urethral/bladder neck slings)
- Artificial urinary sphincter devices

## PERI-URETHRAL BULKING AGENTS

The injection of bulking agents sub-mucosaly in the female urethra is intended to aid continence via apposition of the urethral wall. It is thought to be most useful in the treatment of Blaivas type 3 SUI (intrinsic sphincter deficiency). As well as its use in female SUI, this technique is also reported in males and children and can be carried out under local anesthetia. The advantages of this technique include the low associated morbidity. There is a range of injectable materials from autologous fat, through collagen to manufactured polymers (Teflon, Durasphere, Macroplastique). Autologous fat and PTFE are not widely used due to concerns about their safety profiles.

Both subjective and objective short-term improvement in women with symptoms of SUI was reported in a recent Cochrane review.[9] In addition, Chapple *et al*[10] illustrate that in short-term studies, peri-urethral injection therapy cures or improves 75%. This review of the currently available literature concluded that injection therapy should be considered a first-line treatment for those who have failed conservative measures. The lower complication rate in comparison to more invasive surgical treatments is one factor which favors the use of injection therapy first-line. Despite this, the long-term outcome is questionable and the Cochrane group found no evidence to recommend injection therapy over open surgery in women fit enough for surgery.[9] The use of injection therapy in patients unfit for general anesthesia where short-term outcome is favored was however recommended. Furthermore, two or more treatments may be necessary for the majority of patients.[9]

Longer term studies are necessary before injection therapy can be considered an alternative to open surgery as a durable treatment for SUI. Despite this, patients may favor the low risk of complications and the minimally invasive nature of injection therapy as an initial treatment for SUI prior to considering more invasive open surgery. Complications occur relatively infrequently and are of lower clinical significance than those seen following open surgery.[11] *De novo* urgency, urinary retention, hematuria and particle migration leading to granuloma formation are all reported complications of injection therapy.

## RETROPUBIC SUSPENSION PROCEDURES

Retropubic suspension procedures are mainly intended for the treatment of SUI secondary to urethral hypermobility (types 1 and 2 in the Blaivas classification) and a wide variety of different techniques are available. All have the common underlying principle of elevating and fixing the bladder neck and proximal urethra in a retropubic position to enhance support. The most widely used technique is the Burch colposuspension and this procedure has been used as a gold standard with which to compare newer surgical treatments for SUI.[12] Due to its higher rate of cure and more durable cure rates, Burch colposuspension should be regarded as the standard open retropubic suspension procedure according to a recent review.[13] There is an extensive pool of long-term data regarding both the efficacy and complication rates of this procedure as it has been performed routinely for SUI since the 1960s. Jarvis, in a meta-analysis, reports short-term cure of over 80% both subjectively and objectively.&[14] Excellent long-term results are also available; Alcalay has reported almost 70% cure at 12 years without further decline after that time.[15] These reports highlight the wealth of clinical evidence in support of Burch colposuspension as the standard against which any novel therapies should be compared. Complications however can occur and these include wound problems, extended hospital stay, urinary retention, dysuria, *de novo* detrus or overactivity, urge incontinence, and recurrent UTI's.

Laparoscopic colposuspension has been developed but reports comment that the long-term performance of this procedure is uncertain.[16] Trials had shown a trend toward higher complication rates and longer operating time but highlighted the benefit of less intraoperative blood loss, less postoperative pain, shorter hospital stay, quicker return to normal activities, and shorter duration of catheterization for laparoscopic compared with open colposuspension. Direct comparison studies of laparoscopic versus open colposuspension are surprisingly few and far between.

The Marshall--Marchetti--Krantz colposuspension and needle suspension procedures such as the Raz, Stamey and Peyrera techniques are no longer in routine use for the treatment of SUI.

## SLING AND TAPE PROCEDURES

The earliest reported type of surgery still in use today is the pubo-vaginal sling, and several techniques have been developed using a host of different materials ranging from autologous to cadaveric to biological to synthetic slings. The technique is similar whatever material is used; the pubo-vaginal sling is classically used for type 1 and type 3 SUI. A tissue strip is inserted peri-urethrally via a combination of abdominal and vaginal approaches and is then anchored to the rectus sheath. The sling reinforces the support at the mid-urethral level consistent with current theories of female continence.[17,18] Overall, the results of pubo-vaginal slings are very good. One meta-analysis shows a 12-month success rate of over 80%[14] in both objective and subjective testing. Urinary retention, *de novo* detrusor overactivity, perforation, and erosion are all reported as complications of pubo-vaginal slings. Procedures using bony fixation have been described but this carries a risk of osteitis pubis. Nowadays, the pubo-vaginal sling is being reserved for patients who have refractory SUI or those in whom placement of a synthetic tape would be unwise.

The emergence of low tension sub-urethral tapes has revolutionized the treatment of SUI in the last decade. These techniques are designed to create an anchored pubo-urethral neo-ligament using synthetic mesh to increase the level of mid-urethral support. The theoretical mechanism of action would seemingly benefit only those with type 3 SUI, but several reports exist illustrating their success in all Blaivas classification groups.[19,20] The tension-free vaginal tape (TVT) is the most extensively studied of the low tension sub-urethral tapes and has been the subject of a robust clinical study.[21] Ward and Hilton have reported both short to medium term results of a multi-centre randomized controlled trial comparing TVT with open colposuspension as the gold standard.[21,12] Their most recent report describes an objective 5-year cure rate of 81% in the TVT group versus 90% in the open colposuspension group. This difference was not statistically significant but represents excellent durability for both procedures. The open colposuspension group had higher rates of postoperative enterocele and cystocele and late tape erosions were seen in the TVT group in three cases.[21] This study represents good clinical evidence that TVT provides an equivalent level of efficacy to open colposuspension without the complications associated with open surgery. The low tension sub-urethral tapes carry their own complications such as voiding dysfunction, urinary retention, *de novo* detrusor instability, infection and erosion, all of which can lead to long-term symptoms and should not be underestimated.

Supporting this study is a review by Atherton and Stanton[22] which examines longer term data. 7-year cure rates of over 80% with minimal long-term complications are reported. Potential complications from this technique include injury to blood vessels, abdominal viscera, and urethra, but to date there has been a relatively low number reported. Although voiding disorder after TVT insertion is not negligible, it appears less than with other incontinence procedures.[23] All of these data has led to the rapid emergence of TVT (and other low tension sub-urethral tapes) as a routine treatment for SUI.

The development of the TVT led to trials of a similar low tension sub-urethral tape but using a different route of access; the trans-obturator tape (TOT) again uses mid-urethral tape placement but instead of anchoring supra-pubically like a TVT, it anchors through the obturator foramen. Early trials of multi and single centre studies reported favorable outcomes[20,24] and short-term cure rates of 80-90%. Two methods of inserting a TOT are in existence and both involve the passage of a curved needle through the obturator foramen. The difference is in the direction of penetration. Using the inside-out technique, the needle passes from the sub-urethral position in the midline, while for the outside-in method the needle is passed from a lateral position to emerge through the sub-urethral incision. A recent review by Costa 2004 identified no major differences in efficacy or morbidity between the two techniques of TOT insertion.[25] This has been supported by a recent meta-analysis of direct and indirect comparison trials.[26] Latthe *et al* compared results from over 30 randomized controlled trials and found equal efficacy and no significant difference in complication between either route of insertion.

Longer term and comparative data have recently emerged and a 2009 Cochrane review has examined the evidence for sub-urethral tapes in detail.[27] This report describes the trans-obturator route as less favorable than the retropubic route in terms of objective cure but equivalent in terms of subjective cure. However, there was less voiding dysfunction, blood loss, bladder perforation, and shorter operating time with the trans-obturator route.[27]

Recently, Rechtberger *et al* have reported a randomized trial comparing retropubic versus trans-obturator procedures involving over 500 women.[28] With 18-month follow-up there was no significant difference in clinical efficacy with both routes exhibiting around a 75% cure rate. However 6.5% of retropubic operations were complicated by bladder perforation. Interestingly, the authors point out that the retropubic route may be more efficacious in women with intrinsic sphincter deficiency as defined in this study by an abdominal leak point pressure of 60 cmH_2_ O or less. Longer term studies of TOT outcome are still necessary before it can claim equivalence with a gold standard therapy such as open colposuspension. Despite this, many short-term reports compare favorably with TVT.[28,29] TOT is a promising technique and currently enjoys routine use in the treatment of SUI.

## ARTIFICIAL URINARY SPHINCTER DEVICES

Artificial urinary sphincter (AUS) insertion dates back to the 1970s. The increase in outlet resistance provided by an inflatable cuff around the proximal urethra keeps the patient dry when the cuff is activated and allows bladder emptying when it is not. AUS insertion is often carried out after failure of other surgical treatments, but high continence rates have been reported after primary AUS insertion. Webster *et al* published over 90% continence rates at 2.5 years in women with no previous surgery for SUI.[30] Longer term results are, however, less encouraging. A single-center review of AUS insertion by Thomas *et al*, examined 68 cases and found at a median follow up of 7 years only 37% retained the original sphincter.[31] The reason for removal was infection or erosion in almost half. In this study, a sphincter replacement rate of 16% at 5.5 years for mechanical failure was quoted. A further report from Fulford *et al* provide support for the use of AUS with 10 year follow-up of a series of AUS[32] and describe over 75% continence rates. Despite this, the majority of patients needed supplementary procedures including replacement. It would appear therefore that the use of the AUS for SUI as a primary operation remains controversial due to the risk of complications and common requirement for revision surgery.

## DISCUSSION

The patient with SUI has a large number of surgical options to consider and this review can help with counseling for health care providers within this clinical area. The wealth of data has been accurately recorded and summarized by several well-conducted meta-analyses. The comparison between the proven long-term efficacy but associated risk of surgical complications of open colposuspension versus the less invasive, low morbidity but relatively scarce long term data associated with sub-urethral tapes do not clearly favor either procedure. Equivalence at 5 years has been demonstrated[21] and many urologists believe that longer term efficacy will again be comparable leading to TVT becoming the gold standard surgical procedure for SUI on the basis of equivalent effect for reduced morbidity when compared with open colposuspension.

Indeed in a recently published survey conducted by the International Urogynaecology Association, TVT was the preferred primary incontinence procedure for 68% of responders.[33] Since the introduction of the TVT in 1996, the popularity of this procedure has grown exponentially. The health economic argument for TVT is also a strong one. Cody *et al* have analyzed the cost-effectiveness of various surgical treatments for SUI and identified the reduced hospital stay, complication rate, and recovery time as factors making TVT the most cost-effective treatment for SUI.&[34] The authors do however comment the high levels of treatment success associated with sub-urethral tapes which may lead to increased numbers of women presenting and a larger healthcare burden on society. It is clear that large numbers of women suffer from urinary incontinence and the potential requirement for treatment could be significant.

## CONCLUSION

Although pubo-vaginal slings, bulking agents, retropubic suspensions, and AUS insertion are effective treatments for SUI as described above, their use is limited to individual cases and current practice appears to suggest an overwhelming preference for sub-urethral tapes.[33] The data described above are a strong indication that TVT should be regarded as a proven gold standard surgical treatment for SUI. Newer techniques should be compared to TVT and evaluated in terms of clinical efficacy, patient acceptability, and cost-effectiveness.
